# Aerobic Exercise Alleviates the Impairment of Cognitive Control Ability Induced by Sleep Deprivation in College Students: Research Based on Go/NoGo Task

**DOI:** 10.3389/fpsyg.2022.914568

**Published:** 2022-06-30

**Authors:** Shangwu Liu, Runhong Zhang

**Affiliations:** Department of Physical Education, Luliang University, Luliang, China

**Keywords:** college students, aerobic exercise, sleep deprivation, cognitive control, Go/NoGo task

## Abstract

The purpose of this study was to observe whether aerobic exercise is able to alleviate the impairment of cognitive control ability in college students by sleep deprivation through cognitive control (Go-NoGo task) and blood-based markers. Taking 30 healthy college students (15 males and 15 females) as participants, using a random cross-over design within groups, respectively perform one night of sleep deprivation and one night of normal sleep (8 h). The exercise intervention modality was to complete a 30-min session of moderate-intensity aerobic exercise on a power bicycle. Change in cognitive control was assessed using the Go/NoGo task paradigm; 5-ht and blood glucose contentwere determined by enzyme-linked immuno sorbent assay and glucose oxidase electrode Measurement, respectively. The results showed that sleep deprivation could significantly reduce the response inhibition ability and response execution ability, and significantly reduce the blood 5-ht content (*p*< *0.01*). Thirty minutes of moderate intensity aerobic exercise intervention significantly increased response inhibition ability and response execution ability, significantly increased blood 5-ht content (*p*<*0.01*), and did not change serum glucose levels. Conclusion: An acute aerobic exercise can alleviate the cognitive control impairment caused by sleep deprivation, and 5-ht may be one of the possible mechanisms by which aerobic exercise alleviates the cognitive control impairment caused by sleep deprivation.

## Introduction

With the social progress, the acceleration of the rhythm of life, a more vibrant nightlife and the use of electronic networks and other products, sleep deprivation is a widespread problem in modern society, particularly among young people (Lissak, 2018; Becker et al., 2019). In a cross-sectional survey, more than 70% of young people reported sleeping less than recommended, about 60% of them suffered from severe sleep deficiency (Chaput et al., 2018). Researchers have found that, among young and middle-aged adults, short sleep duration is prevalent in 32% to 39% (Csipo et al., 2021). In addition to the increased risk of chronic somatic diseases (Schwartz et al., 2005; Kasasbeh et al., 2006; Knutson et al., 2006; Taheri, 2006; Zimmerman et al., 2006; Rahmani et al., 2020), short sleep duration is strongly associated with decline in cognitive performance (Chaput et al., 2018; Cousins and Fernández, 2019). Csipo et al. (2021) studies have shown that, in healthy young adults, 24-h sleep deprivation can impair cognitive performance, this included a significant increase in reaction time and a significant decrease in scores on the rapid visual information processing test, which measures sustained attention. Cain et al. (2011) studies of healthy young people have shown that, stroop's task of naming colors, the response time is significantly increased by one night's sleep deprivation. Chan Kwong et al. (2020) studies have shown that, sleep deprivation for 24 h can significantly impair working memory (as determined by the n-back task and rapid visual processing test) in healthy volunteers, the N-back task showed a significant decrease in accuracy, for the rapid visual processing task assessment test, reaction times increased significantly. García et al. (2021) research on healthy college students has shown that, after 24 h of sleep deprivation, the subject's inherent tonic attention, selective and sustained attention (a component of attention) and in cognitive inhibition (a component of executive function) are markedly reduced. Grundgeiger et al. (2014) studies have shown that, sleep deprivation increases failures to carry out intended actions. Freshman in college who presented as subjects after 25 h of sleep deprivation, performance was lower after sleep deprivation and with a more resource- demanding prospective-memory task. Sleep deprivation resulted in slower reaction times and lower accuracy. Riontino and Cavallero (2022) evaluated the impact of one night of sleep deprivation on the efficiency of three attentional networks, following sleep deprivation, reaction times and accuracy were significantly slowed, executive control efficacy significan -tly decreased. Xu et al. (2022) studies have shown that, sleep deprivation for 36 h can significantly decrease working memory (pronunciation-related working memory) levels in healthy males, the accuracy of the articulation-related working memory task was significantly reduced, time to react significantly increased. These findings suggest that, Sleep deprivation appears to affect human cognition at both a global and a unspecific level, which should affect a variety of cognitive tasks.

In many industries such as medicine, transportation, and the military, people usually need to remain awake and perform cognitive tasks in the absence of sleep. Therefore, it is necessary to take measures to counteract the effects of sleep deprivation and to improve cognitive performance during sleep deprivation. Physical exercise involves the stretching of bones by muscles and the consumption of energy, and is a planned and repetitive physical activity designed to promote health, and improve motor skills (Eöry et al., 2021). Studies have shown that physical exercise is an important means to ensure physical and mental health, which can reduce the incidence of physical diseases (such as cardiovascular disease and obesity, etc.) and psychiatric diseases (such as depression and anxiety, etc.) (Mandolesi et al., 2018). In addition, behavioral studies have also shown that physical exercise is able to promote cognitive ability (Li et al., 2017; Elce et al., 2020; Morawietz and Muehlbauer, 2021). From the perspective of exercise groups, physical exercise can promote cognitive function in various groups including underage (Etnier et al., 2014), adult (Karssemeijer et al., 2017), and elderly (Yoon et al., 2018). From the perspective of exercise characteristics, different types (e.g., endurance training, coordination training, and stretching training), intensity, and duration of physical exercise can promote cognitive ability (Mollinedo Cardalda et al., 2019). From the perspective of training effect, physical exercise can also improve different types of cognitive function (such as visual memory, auditory memory, problem-solving ability and cognitive control ability, etc.), and the promoting effect can be maintained for a period of time after the end of exercise (Donnelly et al., 2016; Song et al., 2018; Zhou et al., 2022). Interestingly, not only long-term exercise, but even acute exercise has positive benefits on higher-order cognitive functions such as concentration, memory, and even executive function (Niedermeier et al., 2020; Cantelon and Giles, 2021). Animal studies have shown that exercise protects the memory from acute but not chronic sleep deprivation (Roig et al., 2022). Sahin et al. (2021) studied protective effects of mild treadmill exercise on acute sleep deprivation rats, the results indicate that, an acute sleep-deprivation period of 48 h impairs long-term spatial memory significantly. The adverse effects of acute sleep deprivation on memory can be mitigated by mild, regular treadmill exercise. Zagaar et al. (2012) studies have shown that, regular exercise program prevents the sleep deprivation induced impairments in short-term memory and early phase long-term potentiation (LTP) by preventing deleterious changes in the basal and post-stimulation levels of P-CaMKII and BDNF due to sleep deprivation. Saadati et al. (2015) investigate the effects of physical exercise on cognitive functions of female rats following paradoxical sleep deprivation, the results indicate that, 4 weeks of treadmill running alleviated the paradoxical sleep deprivation (PSD)—induced learning and memory impairments in both intact and ovariectomized groups. In humans, it is not known whether exercise can protect memory from the effects of sleep loss. Scott et al. (2006) researched the effect of a 30-h of sleep deprivation and intermittent physical exercise on cognitive, motor performance and mood, the results indicate that, when compared to those who are deprived of sleep alone, individuals that performed 5 h of intermittent moderate exercise during 30 h of sleep deprivation were more susceptible to negative. So, whether acute, a single bouts of exercise improve cognitive control impairment due to sleep deprivation? This needs to be confirmed by further studies.

A study compared older, poor memory rats without training to rats with improved memory after training, the expression of 5-ht and receptors associated with it differs significantly in the relevant brain tissues, this suggests that 5-ht and its receptors regulate learning and memory in the brain (Elmenhorst et al., 2012). As reviewed by Cools et al. (2008), in the OFC, serotonergic activity correlates with levels of response inhibition and performance in reversal learning. Marmosets with low levels of 5-ht were unable to inhibit prepotent responses (Walker et al., 2006). As well as disrupting inhibitory control, 5-ht depletion in the orbitofrontal cortex leads to substantial deficits in reversal learning in monkeys and rats (Clarke et al., 2007; Lapiz-Bluhm et al., 2009). When healthy volunteers are acutely depleted of tryptophan, the same effect is observed on human performance in reversal learning tasks (Murphy et al., 2002). In this study, we investigated whether acute aerobic exercise can alleviate the cognitive impairment caused by sleep deprivation, and 5-ht may be one of the possible mechanisms by which acute aerobic exercise alleviates the cognitive control impairment caused by sleep deprivation.

## Study Subjects and Methods

### Study Subjects

Thirty college students (15 males and 15 females), aged 19–24 years (mean age 22.3 ± 1.3 years; body mass index 22.5 ± 0.5 kg/m^2^), were recruited from Lu Liang University by means of Internet and posting notices as study subjects. This study was reviewed and approved by the Ethics Committee of Luliang University. All study subjects were informed of the purpose of this study, volunteered to participate in the experiment, and signed an informed consent form.

Screening criteria for the study subjects:(1) Individuals with normal physical mobility in the Physical Activity Questionnaire (PAR-Q questionnaire) survey; (2) Individuals with normal intelligence as assessed by the Mini-Raven's Tweet Intelligence Scale; (3) Individuals with normal uncorrected or corrected visual acuity; (4) No physical, neurological, or psychiatric disorders, no recent medication; (5) Have good sleep habits, no staying up late recently; (6) score no more than 22 on the Morningness–Eveningness scale (Horne and Ostberg, 1976); (7) have no symptoms associated with sleep disorders; (8) have no history of any psychiatric or neurologic disorders; (9) No contraindications to exercise. In addition, individuals were excluded based on the following criteria: vegetarian, current efforts to lose weight (e.g., dieting), irregular meal timing habits, irregular sleep/wake habits, and habitual smoking, drinking and coffee.

### Study Design and Procedure

According to the purpose of the study, all subjects needed to complete two parts of the experimental tasks of total sleep deprivation and aerobic exercise intervention. The former aims to investigate the changes in cognitive control ability of subjects in Go/NoGo task after sleep deprivation, and the latter aims to investigate the intervention effect of aerobic exercise on cognitive control ability of subjects after sleep deprivation.

### Sleep Depravation

**Figure 1:**
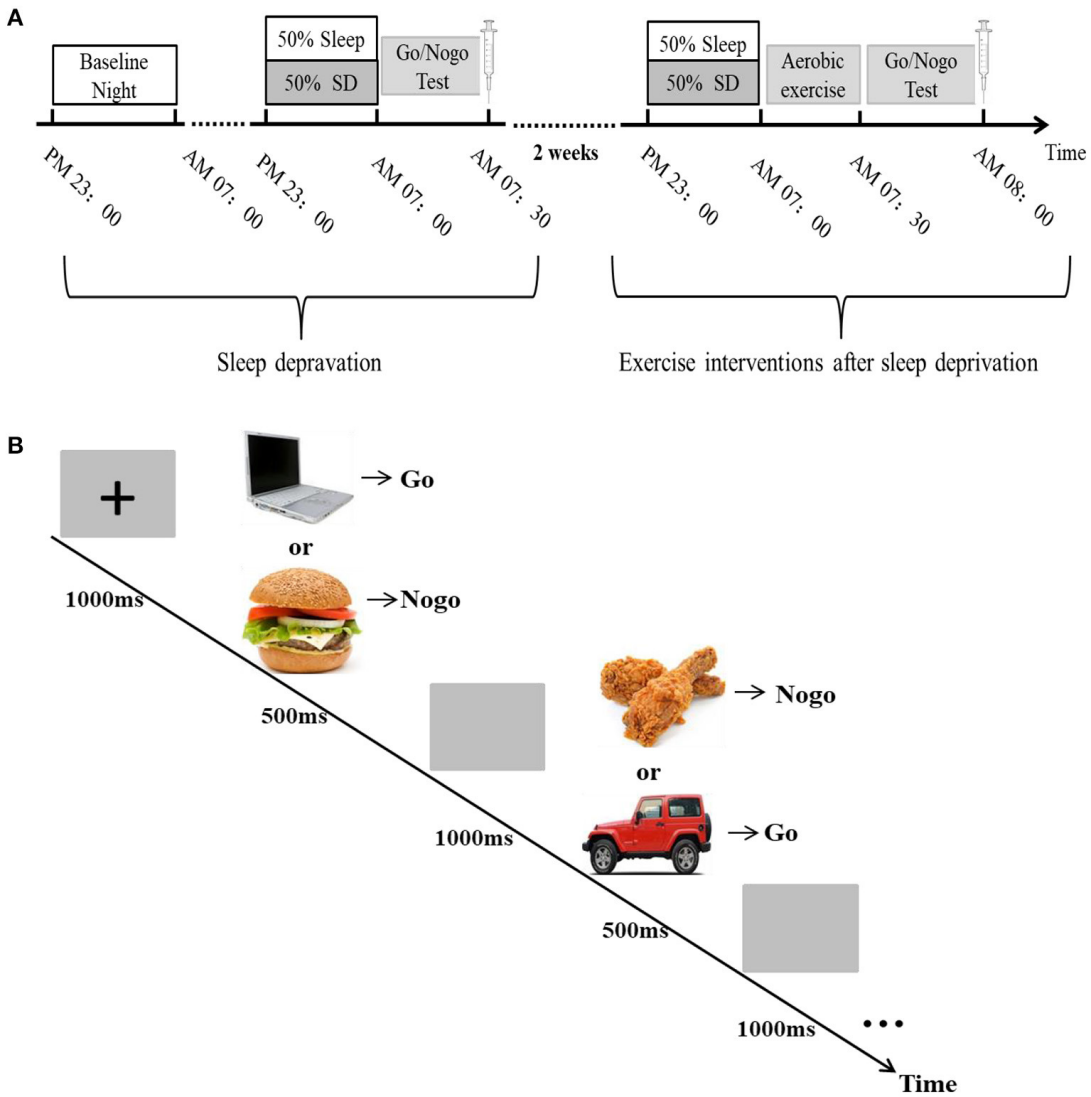
Flow chart of test procedures. **(A)** A randomized crossover experimental design process. All subjects are required to participate in a sleep deprivation and a normal sleep experiment, with an interval of 2 weeks. The sleep intervention time is PM23:00-AM07:00. Sleep in the basal state (baseline night) was performed once before the experiment, and a Go/NoGo experiment and blood sample collection between 07:00 and 08:00 after the sleep intervention. **(B)** Go/NoGo experimental flowchart.

A randomized, balanced crossover design was used. Thirty subjects were randomly divided into sleep deprivation group (15 subjects) and normal sleep group. Each group had to participate in the experiment of sleep deprivation and normal sleep, with an interval of 2 weeks. The time span of both the normal sleep and sleep deprivation experiments ranged from 23:00 nights to 07:00 the next day (Figure 1). For each condition, participants came to the lab two evenings before the experimental morning, and stayed in the lab until the experiments were conducted, and got a standard meal. Participants were blinded to the experimental condition (i.e., Sleep vs. TSD) until being informed of the respective condition 90 min in advance of intervention onset. During normal sleep (23:00–07:00), the light of the laboratory was turned off, and the sleep status of the subjects was monitored using a polysomnography (Embla Flaga hf, Reykjavik, Iceland), and the sleep stages were distinguished according to the criteria determined by Hobson (Hobson, 1969). During sleep deprivation (23:00–07:00), all subjects had to remain awake at all times, and they could see a computer, book, play games, and walk freely in the laboratory, but could not eat any food and could not sleep. In the morning after the sleep intervention (07:00–08:00), the cognitive control ability of the subjects, as well as fasting glucose and 5-ht content, were measured, respectively (Figure 1A).

### Aerobic Exercise

The aerobic exercise intervention experiment was started 2 weeks after the end of the total sleep deprivation experiment. In the experiment, 30 subjects were randomly divided into aerobic exercise intervention group (15 subjects) and sedentary rest group after completing a sleep deprivation treatment. In order to ensure that the order of exercise treatment and sedentary rest treatment is balanced between subjects, this part of the experiment, we are still using an intra-group crossover design, each subject is subjected to aerobic exercise and sedentary rest after sleep deprivation, with an interval of 2 weeks. Among them, the aerobic exercise group needed to complete a 30-min aerobic exercise intervention after sleep deprivation, and cognitive control ability was measured immediately after exercise, 30 min after exercise and 1 h after exercise, while venous blood was collected immediately after exercise and at 1 h after exercise to test fasting blood glucose and 5-ht. Cognitive function tests and blood sampling times in the sedentary rest group were consistent with those in the exercise group (see Figure 1A).

Aerobic exercise protocol Reference (Weinstock et al., 2012), 30 min of moderate- intensity aerobic exercise on a MONARK 834 power bicycle (Swedish). Exercise intensity was graded using the American College of Sports Medicine Aerobic Exercise Intensity Grading Criteria for Healthy Adults (American College of Sports Medicine, 2006). During exercise, the resistance range of the power bicycle was between 0 and 150 W, and the bicycle rhythm was ≥30 r/min. Before the formal exercise intervention, the subjects performed a warm-up exercise for 5 min in the power car, followed by continuous exercise for 30 min when the exercise load reached 60–69% of the individual's maximum heart rate, with a maximum heart rate = 220 – age. Exercise intensity was monitored using a Polar table and an RPE table throughout exercise (Borg, 1985), and heart rate and RPE were recorded every 2 min.

### Cognitive Control Ability Tests

In this study, we used the Go/NoGo task paradigm associated with food pictures and non-food pictures to evaluate the cognitive control ability of the subjects (Verbruggen et al., 2014; Eöry et al., 2021). Non-food and food-related words represented Go and no-Go trials, respectively. The experimental program was written in E-prime, and at the beginning of the experiment, a black “+” sign with a duration of 1,000 ms was first presented in the center of the computer display screen as the fixation point, followed by a Go (appearance of a non-food related pichture) or Nogo (appearance of food-related word) trial, each trial presentation time was 500 ms. The subjects were required to respond to the Go trial of non-food and not to the food-related Nogo trial, and entered the next trial after a 1,000 ms gray screen, as shown in the experimental flow. A total of 160 pictures appeared in the 4-min task, where, food word occurrence constituted a quarter of the total words in the task, and these pictures are mainly composed of some foods they are familiar with (e.g., hamburgers, noodles, fried chicken legs, etc.), Non-food pictures are some items that subjects often see (e.g., tables, computers, cars, etc.). The subjects were asked to press the keyboard button using their dominant hand both quickly and accurately when the picture appeared. There are 5 mission versions available as balanced crossovers, and when one group uses one version of the task, the other uses another. A training session was performed to ensure that the participants understood the task. All of the participants had hit rates that were 90% or above before the formal test (Figure 1B).

### Biochemical Analyses

Plasma glucose analysis carried out on a chemistry analyzer (Architect C16000, Abbott Laboratories). 5-ht content was detected by enzyme-linked immunosorbent assay, and the kits were from Tongwei Reagent (Shanghai) Co., Ltd.

### Data Analysis

The outcome variables of Go/NoGo task performance included, the key accuracy rate (hit rate) of go trials, the no-key rate (omission errors) of go trials, the response time (RT) for responses and the intra-individual coefficient of variation (ICV), and the key rate of NoGo trials. Among them, hit rate, response time, ICV, and omission errors of go trials reflect response execution ability in cognitive control. Commission errors are defined as the ratio of inhibition failures when subjects face NoGo stimuli (Cedernaes et al., 2014) and can reflect response inhibition ability. Response time was defined as the time between Go gignal appearance and correct key press response (Vainik et al., 2013). Intraindividual coefficient of variation (ICV) is the variability of subjects' reaction time in go trials (Castellanos et al., 2005; Chuah et al., 2006).

Statistical analysis of all data was performed using SPSS 17.0 software. In order to examine the effects of sleep deprivation on cognitive control ability and blood biochemical parameters of the subjects, first by independent sample *t*-test, after verifying that the sequential effect of the crossover design within the group was negative, the normal distribution of the data was examined using Kolmogorov–Smirnov's test of normality, and after verifying that the data were normally distributed, cognitive control ability (commission errors, response time, hit rate, and ICV) was analyzed using a 2-factor repeated measures analysis of variance with 2 (group: sleep and TSD) × 2 (gender: male and female) × 2 (task conditions: Go and Nogo conditions), and blood biochemical parameters (glucose and 5-ht) were analyzed using a 2-factor repeated measures analysis of variance with 2 (group: sleep and TSD) × 2 (gender: male and female). In order to verify the effect of exercise intervention on the cognitive control ability and blood biochemical indicators of the subjects after sleep deprivation, the processing factors (exercise and rest) were used as the variables within the group, and the repeated measures analysis of variance was further used to analyze the processing factors (exercise and rest), time factors (immediately after exercise, 30 min after exercise and 1 h after exercise) and their interaction effects on cognitive control (hit rate, reaction time, omission errors, commission errors and ICV). Throughout the analysis, the Greenhouse Geisser method was used to correct degrees of freedom and *p*-values for statistics that did not meet the spherical test, and Bonferroni was used for *post-hoc* comparisons. All data were expressed as mean and standard deviation, and the level of significance was set at *p*< *0.05*.

## Results

### Effect of Sleep Deprivation on Cognitive Control Ability of College Students

**Figure 2:**
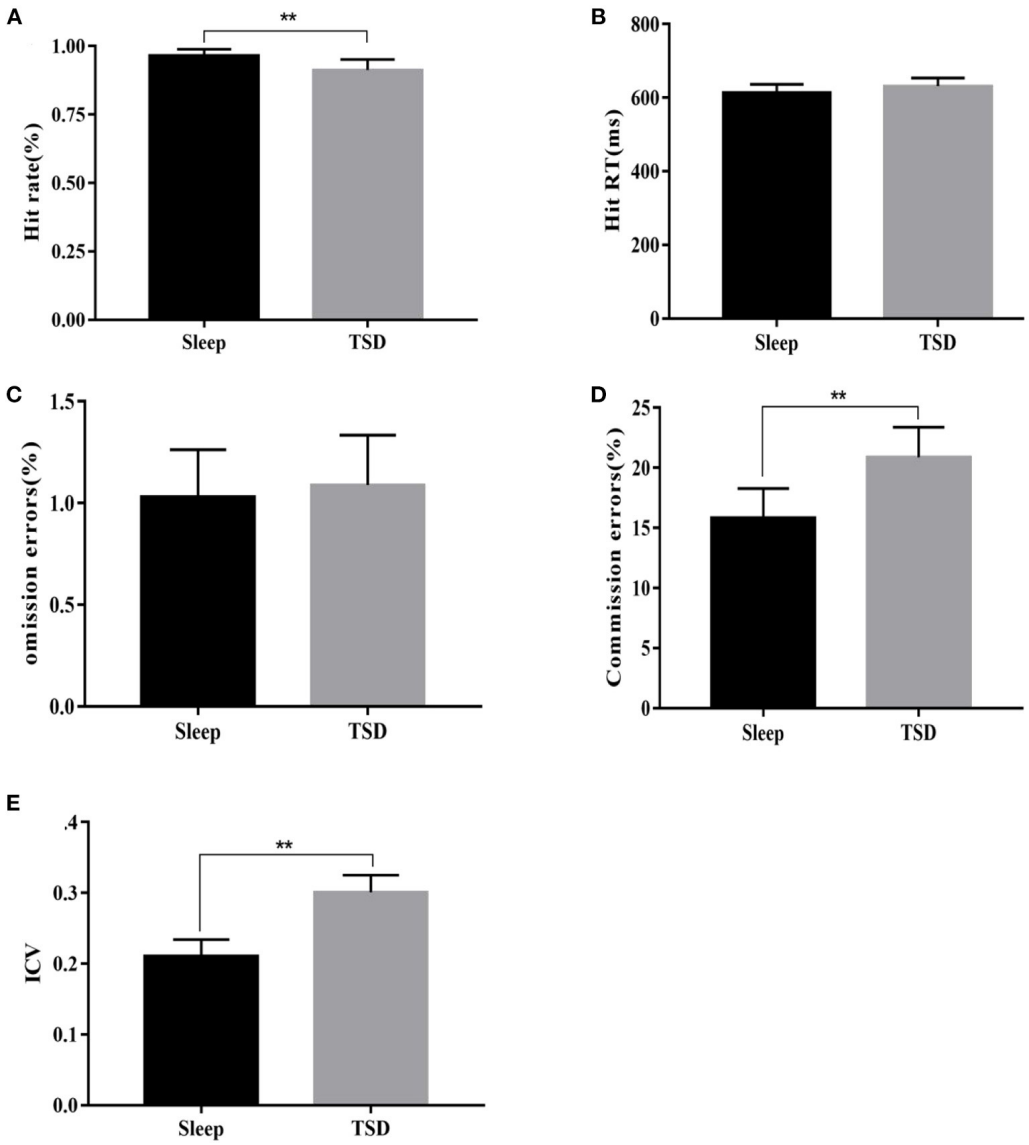
Comparison of changes in various indicators of Go/Nogo task under sleep deprivation and normal sleep conditions. **(A)** Change in hit rate, **(B)** Change in RT, **(C)** Change in the number of omission errors, **(D)** Change in the number of commission errors, and **(E)** Change in ICV. **p <0.01.

After using a 2 (group: sleep and TSD) × 2 (gender: male, female) 2-factor repeated measures analysis of variance on the sleep deprivation experimental data of the first part (Figure 2), it was found that compared with normal sleep, the subjects had a significantly lower hit rate in the face of Go signal stimuli after TSD (0.97 ± 0.01 vs. 0.93 ± 0.03% of non-food Pictures), the main effect of sleep was significant [*F*_(1, 58)_ = 56.84, *p* = 0.0001, η^2^ = 0.50], the main effect of gender was not significant [*F*_(1, 58)_ = 0.98, *p* = 0.33], and the interaction between the two was also not significant [*F*_(1, 58)_ = 1.22, *p* = 0.28]. Compared with normal sleep, the response time (RT) of subjects tended to increase after TSD (613.29 ± 16.58 vs. 628.81 ± 20.72 ms, *p* > 0.05), but the main effect of sleep was not significant [*F*_(1, 58)_ = 0.82, *p* = 0.19], nor was the main effect of gender [*F*_(1, 58)_ = 0.73, *p* = 0.31], and the interaction between gender and sleep was not significant [*F*_(1, 58)_ = 1.09, *p* = 0.07]. The number of omission errors tended to increase, but did not change significantly (1.07 ± 0.17 vs. 1.14 ± 0.14% of total non-food Pictures, *p* > *0.05*), the main effect of sleep was not significant [*F*_(1, 58)_ = 1.10, *p* = 0.14], nor was the main effect of gender [*F*_(1, 58)_ = 0.26, *p* = 0.6138], nor was the interaction between the two [*F*_(1, 58)_ = 0.67, *p* = 0.841]. The number of commission errors was significantly increased compared with the normal sleep group (15.86 ± 2.41% vs. 20.87 ± 2.49% of total food Pictures), the main effect of sleep was significant [*F*_(1, 58)_ = 59.5, *p*<*0.0001*, η^2^ = 0.51], the main effect of gender was not significant [*F*_(1, 58)_ = 0.26, *p* = 0.6138], and the interaction between the two was also not significant [*F*_(1, 58)_ = 0.44, *p* = 0.9342]. ICV significantly increased in subjects after TSD (0.21 ± 0.03 vs. 0.30 ± 0.02, *p* <0.05), the main effect of sleep was significant [*F*_(1, 58)_ = 192.99, *p*<*0.0001*, η^2^ = 0.77], the main effect of sex was not significant [*F*_(1, 58)_ = 0.12, *p* = 0.73], and the interaction between sleep and sex was not significant [*F*_(1, 58)_ = 0.04, *P* = 0.85]. The above results showed that sleep deprivation could seriously impair the response inhibition ability of the subjects, and there was no difference in this impairment between genders.

### Effect of Sleep Deprivation on Blood Glucose and 5-ht Levels in College Students

**Figure 3:**
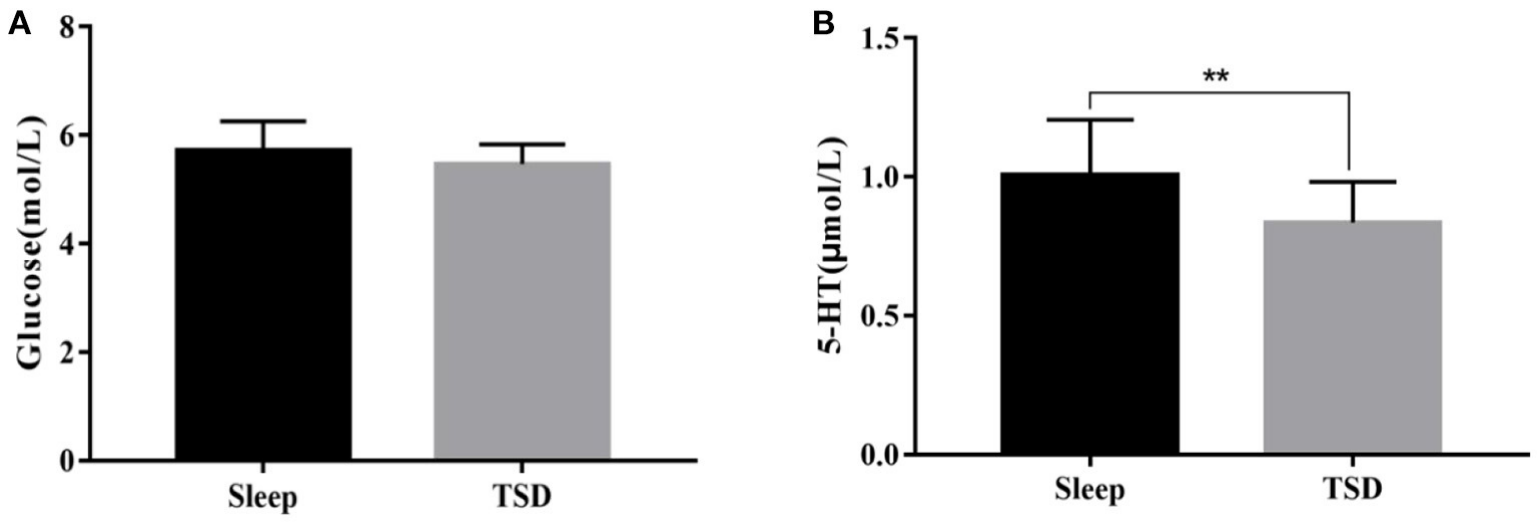
Comparison of fasting blood glucose and 5-ht content changes under sleep deprivation and normal sleep conditions. **(A)** Change in the content of glucose and **(B)** Change in the content of 5-HT. **p <0.01.

There was no significant change in glucose content in the peripheral blood of subjects in the TSD group compared with normal sleep status (5.47 ± 0.36 VS. 5.62 ± 0.54 mmol/L), no significant main effect of sleep [*F*_(1, 58)_ = 0.99, *p* = 0.1633], no significant main effect of gender [*F*_(1, 58)_ = 0.16, *p* = 0.69], and no significant interaction between gender and sleep processing [*F*_(1, 58)_ = 0.09, *p* = 0.76]. After TSD, 5-ht content was significantly reduced (1.03 ± 0.22 vs. 0.83 ± 0.17 μmol/L), the main effect of sleep was significant [*F*_(1, 58)_ = 14.40, *p*<*0.01*, η^2^ = 0.2], the main effect of gender was not significant [*F*_(1, 58)_ = 0, *p* = 0.95], and the interaction between gender and sleep processing was also not significant [*F*_(1, 58)_ = 0.41, *p* = 0.53]. As a result, TSD had no significant effect on fasting blood glucose content in subjects, but it could reduce 5-ht content in the blood, and there was no difference between genders in this effect (Figure 3).

### Effect of Exercise Intervention on Response Inhibition Ability in College Students With Sleep Deprivation Time-Course Effect

The results of the first part of the experiment showed that TSD could indeed impair the cognitive control ability of the subjects in the face of food signals. On this basis, we further applied a 30-min aerobic exercise intervention to subjects with TSD, and compared the behavioral performance of subjects in the Go-Nogo task immediately after exercise (0 min), 30 min after exercise, and 1 h after exercise, and found that hit rate had a significant main effect of exercise processing immediately after exercise [F (1, 58) = 56.96, *P*<*0.01*, η^2^=0.5], 30 min after exercise [*F*_(1, 58)_ = 50.48, *p* <0.01, η^2^ = 0.47], and 1 h after exercise [*F*_(1, 58)_ = 46.31, *p* <0.01, η^2^ = 0.44], that is, 1 h after the end of aerobic exercise, the hit rate of exercise treatment was higher than that of sedentary rest treatment (0 min after exercise treatment−0 min after rest treatment = 9.51%, *p* = 0.008; 30 min after exercise treatment−30 min after rest treatment = 8.1%, *p* = 0.008; 1 h after exercise treatment−1 h after sedentary treatment = 6.51%, *p* = 0.006). The main effect of hit rate on time after movement processing was not significant [*F*_(2, 177)_ = 1.03, *p* = 0.46].

RT had an exercise treatment effect on all three time periods: immediately after exercise [*F*_(1, 58)_ = 58.98, *p*<*0.01*, η^2^=0.5], 30 min after exercise [*F*_(1, 58)_ = 40.13, *p*<*0.01*, η^2^ = 0.41] and 1 h after exercise [*F*_(1, 58)_ = 30.61, *p* = 0.0017, η^2^ = 0.35], that is, 1 h after the end of aerobic exercise, RT was lower than that in the sedentary rest group (0 min after exercise treatment−0 min after rest treatment = −52.21 ms, *p*<*0.01*; 30 min after exercise treatment−30 min after rest treatment = −42.01 ms, *p*<*0.01*; 1 h after exercise treatment−1 h after rest treatment = −30.1 ms, *p*<*0.01*). There was also a time main effect of RT after exercise treatment [*F*_(2, 177)_ = 8.24, *p*<*0.01*, η^2^ = 0.09], that is, RT immediately after exercise was shorter than that at 30 min and 1 h after exercise (0 min after exercise treatment = −17.28 ms, *p*<*0.01*; 0 min after exercise treatment = −28.69 ms, *p*<*0.01*), and there was also a significant difference at 30 min after exercise compared with 1 h after exercise (30 min after exercise treatment −1 h after exercise treatment = −12.06 ms, *p*<*0.01*). There was no interaction between exercise processing and processing time [*F*_(2, 177)_ = 1.01, *p* = 0.225]. The number of omission errors did not have an exercise treatment effect immediately after exercise [*F*_(1, 58)_ = 1.09, *p* = 0.24], 30 min after exercise [*F*_(1, 58)_ = 0.89, *p* = 0.59], and 1 h after exercise [*F*_(1, 58)_ = 0.93, *p* = 0.36]. The main effect of time after exercise treatment was also not significant [*F*_(2, 177)_ = 1.53, *p* = 0.26], that is, omission errors did not change significantly during the three time periods immediately after exercise, 30 min after exercise, and 1 h after exercise (all *p* > *0.05*). There was also no interaction between movement processing and processing time [*F*_(2, 177)_ = 0.87, *p* = 0.511]. The number of commission errors had a main effect of exercise treatment immediately after exercise [*F*_(1, 58)_ = 46.97, *p*<*0.01*, η^2^ = 0.45], 30 min after exercise [*F*_(1, 58)_ = 40.13, *p*<*0.01*, η^2^ = 0.41], and 1 h after exercise [*F*_(1, 58)_ = 38.61, *p*<*0.01*, η^2^ = 0.4], that is, at 1 h after aerobic exercise, the errors in the exercise treatment group were lower than those in the sedentary rest treatment group (0 min after exercise treatment−0 min after rest treatment = −6.2%, *p*<*0.01*; 30 min after exercise treatment−30 min after rest treatment = −6.17%, *p*<*0.01*; 1 h after exercise treatment−1 h after rest treatment = −5.96%, *p* <0.01). The commission also had a time main effect after exercise treatment [*F*_(2, 177)_ = 5.56, *p*<*0.01*, η^2^ = 0.06], that is, the errors immediately after exercise were lower than those at 30 min after exercise and 1 h after exercise (0 min after exercise treatment−30 min after exercise treatment = −1.38%, *p*<*0.05*; 0 min after exercise treatment−1 h after exercise treatment = −2.34%, *p*<*0.01*), and there was no significant difference between 30 min after exercise and 1 h after exercise (30 min after exercise treatment−1 h after exercise treatment = −0.58%, *p* = 0.374). There was no interaction between movement processing and time Movement processing × time: [*F*_(2, 177)_ = 1.37, *p* = 0.2566].

**Figure 4:**
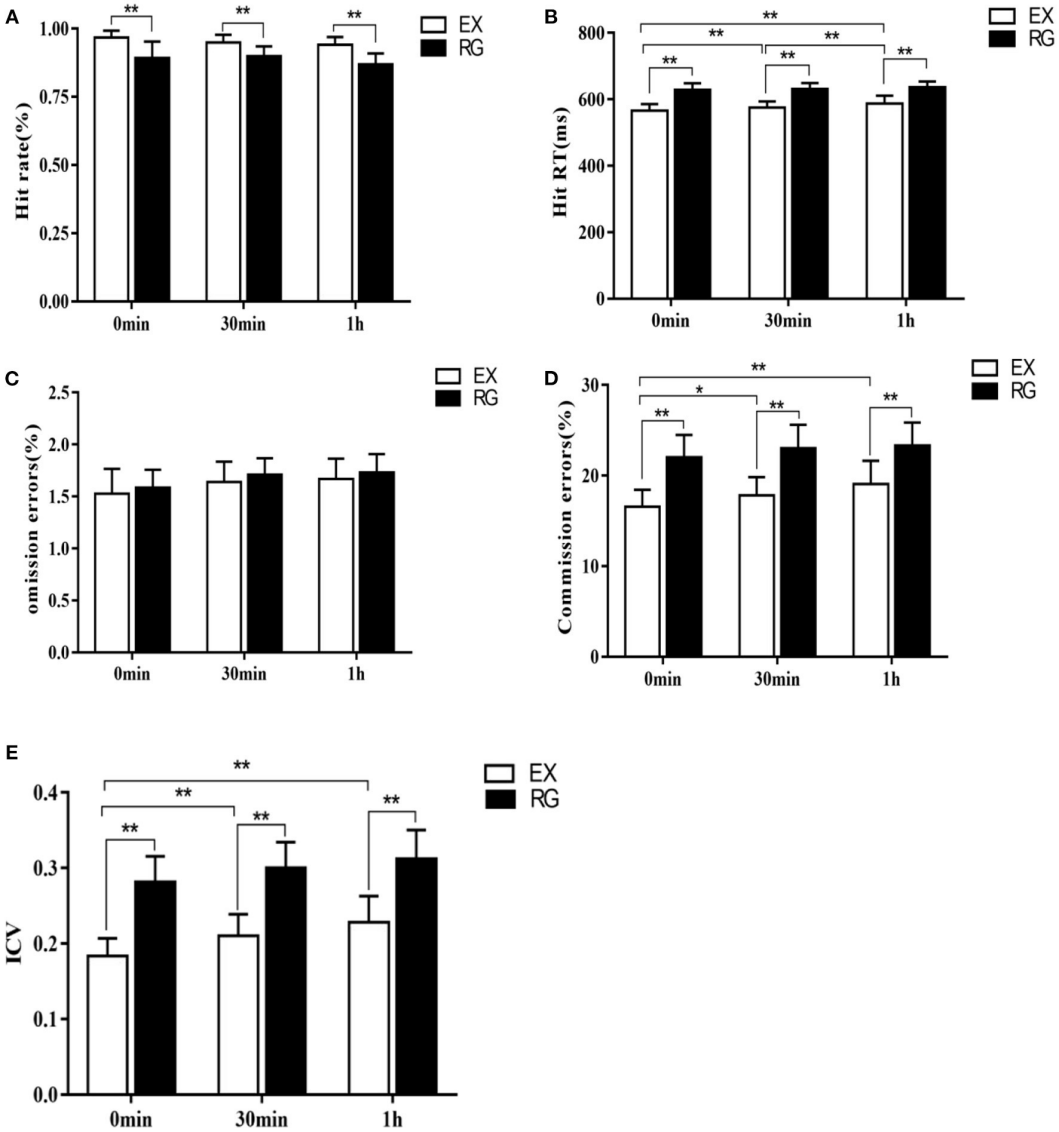
Comparison of changes in each index of Go/Nogo task at different time points after exercise intervention. **(A)** Change in hit rate, **(B)** Change in RT, **(C)** Change in the number of omission errors, **(D)** Change in the number of commission errors, and **(E)** Change in ICV. *p <0.05, **p <0.01.

ICV had a main effect of exercise treatment at all three time points: immediately after exercise [*F*_(1, 58)_ = 177.29, *p*<*0.01*, η^2^ = 0.75], 30 min after exercise [*F*_(1, 58)_ = 159.58, *p*<*0.01*, η^2^ = 0.73] and 1 h after exercise [*F*_(1, 58)_ = 148.69, *p* <0.01, η^2^ = 0.72], that is, at 1 h after the end of exercise, ICV was lower than that in the sedentary rest treatment group (0 min after exercise treatment−0 min after rest treatment = −0.092, *p*<*0.01*; 30 min after exercise treatment−30 min after rest treatment = −0.078, *p*<*0.01*; 1 h after exercise treatment−1 h after rest treatment = −0.064, *p*<*0.01*). There was a time main effect of ICV at three time points after exercise treatment [*F*_(2, 177)_ = 9.01, *p*<*0.01*, η^2^ = 0.09], that is, ICV immediately after exercise was significantly lower than 30 min after exercise and 1 h after exercise (0 min after exercise treatment = −0.021, *p*<*0.01*; 0 min after exercise treatment = −0.033, *p*<*0.01*), and there was no significant difference between 30 min after exercise and 1 h after exercise (30 min after exercise treatment −1 h after exercise treatment = −0.009, *p* = 0.079). There was no interaction between exercise processing and processing time [*F*_(2, 177)_ = 1.28, *p* = 0.089] (Figure 4).

### Effect of Exercise Intervention on Blood Glucose and 5-ht in College Students With Sleep Capture Time-Course Effect

**Figure 5:**
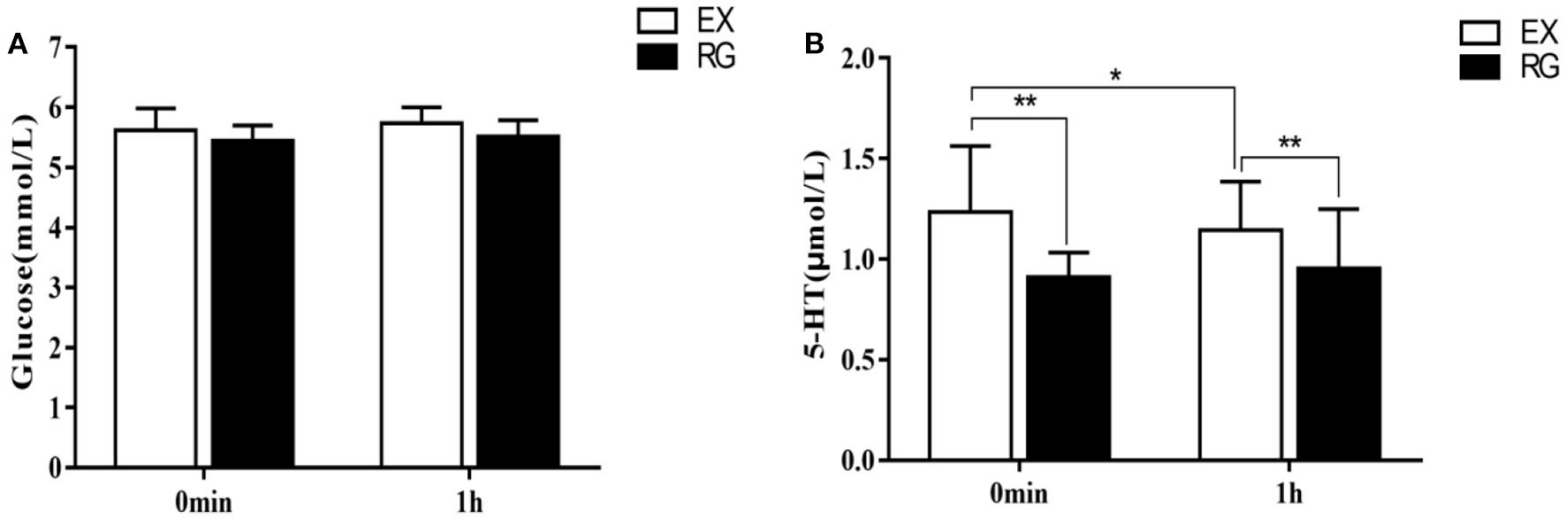
Comparison of changes in fasting blood glucose and 5-ht content after exercise intervention. **(A)** Change in the content of glucose and **(B)** Change in the content of 5-HT. *p <0.05, **p <0.01.

The results of repeated measures analysis of variance of fasting glucose content immediately after exercise and 1 h after exercise showed that there was no main effect of exercise treatment immediately after exercise [*F*_(1, 58)_ = 0.99, *p* = 0.24] and 1 h after exercise [*F*_(1, 58)_ = 0.95, *p* = 0.19], that is, there were no significant changes in fasting blood glucose content immediately after exercise and 1 h after exercise compared with the sedentary rest group (5.49 ± 0.33 vs. 5.33 ± 0.32 mmol/L, *p* > 0.05; 5.52 ± 0.53 VS. 5.42 ± 0.42 mmol/L, *p* > *0.05*). In addition, there was also no time main effect on the fasting blood glucose content of the subjects after exercise [*F*_(1, 118)_ = 2.91, *p* = 0.09], that is, there was no significant change in the fasting blood glucose of the subjects immediately after exercise and 1 h after exercise (0 min after exercise treatment = −0.05 mmol/L, *p* > *0.05*). There was no interaction between exercise processing and processing time [*F*_(1, 118)_ = 0.99, *p* = 0.257]. The results of repeated measures analysis of variance of 5-ht content in the peripheral blood of the subjects immediately after exercise and 1 h after exercise showed that there was a main effect of exercise treatment immediately after exercise [*F*_(1, 58)_ = 25.53, *p* = 0.0004, η^2^ = 0.31] and 1 h after exercise [*F*_(1, 58)_ = 26.89, *p*<*0.01*, η^2^ = 0.32], that is, the 5-ht content of the subjects immediately after exercise and 1 h after exercise was significantly increased compared with the sedentary rest treatment group (0 min after exercise treatment = 0.37 μmol/L, *p*<*0.01*; 1 h after exercise treatment−1 h after rest treatment = 0.24 μmol/L, *p*<*0.01*). There was also a time main effect on 5-ht levels in subjects after exercise [*F*_(1, 118)_ = 1.12, *p* = 0.2928], that is, 5-TH content was significantly higher immediately after exercise treatment than 1 h after exercise (0 min after exercise treatment = 0.11, *p*<*0.05)*. There was no interaction between exercise processing and processing time [*F*_(1, 118)_ = 1.14, *p* = 0.154] (Figure 5).

### Correlation Analysis of Reactivity Inhibition Ability With Blood Glucose and 5-ht in College Students After Exercise Intervention

The results of Pearson's simple correlation analysis showed (Table 1) that there was no significant correlation between cognitive control function-related parameters and blood glucose changes in college students after exercise intervention (*p* > *0.05*). The results of simple correlation analysis showed that commission errors and ICV showed a highly significant negative correlation with 5-ht content changes (*p*<*0.01*). The results suggest that the decrease of 5-ht content after sleep deprivation may be an important factor leading to the loss of some cognitive control functions, while aerobic exercise intervention may reverse the loss of cognitive control caused by sleep deprivation by increasing 5-ht content in peripheral blood.

**Table 1:** Correlation analysis between cognitive control function and blood parameters.

	**Hit rate**	**hit RT**	**Omissions errors**	**Commission errors**	**ICV**
Glu	−0.107	0.124	−0.099	−0.134	0.144
5-ht	0.454^**^	0.104	−0.107	−0.521^**^	−0.475^**^

**p <0.01.

## Discussion

The study found that one night of sleep deprivation is sufficient to impair the execution of cognitive control and the inhibition of cognitive control in normal adults. This impairment of cognitive control ability was significantly alleviated by 30 min of aerobic exercise after sleep deprivation. This beneficial effect can last for about an hour after exercise.

### Impaired Cognitive Control After Sleep Deprivation

Cognitive control is an ability to perform behavior according to rules, purposes, or intentions, two main parts are involved: execution and inhibition of reactions (Davranche and McMorris, 2009), this is a core component of cognitive function. In the first part of this study, the Go-Nogo task was used to assess the cognitive control function of 30 healthy adults after one night of complete sleep deprivation, findings revealed that, all subjects after total sleep deprivation, in the face of Nogo food picture stimuli, there was a significant increase in commission errors compared to the normal sleep group, the results indicate that sleep deprivation impairs the ability to inhibit response. In addition, compared with the normal sleep group, the sleep deprived group's Hit rate was significantly lower in response to go non-food picture stimulation, ICV increases significantly, the number of omission errors and RT tended to increase but there was no significant difference, suggesting that sleep deprivation can also impair the response execution ability of subjects to some extent. It has also been found that, sleep deprivation can increase impulsivity in response inhibition tasks, reduce response inhibition ability (Chuah et al., 2006), increase food intake (Herzog et al., 2012), and enhance rewarding effects in the central nervous system evoked by eating (Benedict et al., 2012). In this study, subjects' attention bias to food stimuli after sleep deprivation made them more likely (although inaccurate) to respond in the face of Nogo food picture stimuli, which in combination with lower executive function further weakened response inhibition, ultimately leading to an increase in commission errors. The velocity-correct rate theory suggests that increasing speed impairs the correct rate. However, this study found that there was no significant change in reaction time while the accuracy was reduced in the sleep deprivation state, and the results suggested that these two distinct automatic responses and stop responses may depend on different aspects of the attention system. In this study, after sleep deprivation, subjects made more commission errors, rather than omission errors, possibly because they sacrificed accuracy in pursuit of speed. Because the Go/NoGo commission task requires accurate inhibition of unrelated responses and continuous error monitoring, the commission errors were significantly increased in this experiment, which may also be related to the inability of subjects to effectively concentrate after sleep deprivation, resulting in reduced recognition of target stimuli (Koslowsky and Babkoff, 1992), and the difficulty in detecting Nogo stimuli or in inhibiting impulsivity caused by Nogo food image stimuli (Jin et al., 2015). Studies by Chee et al. (2006) and Mu et al. (2005) (also confirmed that changes in cognitive function after sleep deprivation have a significant neural basis, and there is a direct relationship between the degree of brain activation and the level of cognitive function. During waking at rest, the activity of the ventrolateral PFC, and the degree of reduced inhibition efficiency after sleep deprivation (Wager et al., 2005). Successful response inhibition (stop) requires activation of lateral prefrontal cortex regions (Konishi et al., 1998), errors of commission are associated with activation of the anterior cingulate cortex and medial frontal gyrus (Hester et al., 2004), the anterior cingulate gyrus plays an important role in conflict monitoring (Braver et al., 2001), while the ventral part of the prefrontal cortex plays an important role in sustained attention control (Egner and Hirsch, 2005) and inhibition of unrelated response processes (Fujimoto et al., 2020). The response to food images in the anterior brain region of the right cingulate cortex is enhanced after total sleep deprivation (Benedict et al., 2012). The study by Chuah et al. (2006) also confirmed that after sleep deprivation, the variability (ICV) of the subjects in the Go/NoGo task was significantly increased, the correct rate (hit rate) was significantly reduced, and the reduced inhibition efficiency was related to the decreased activity in the ventral and anterior regions of the prefrontal cortex (Pfc). Therefore, the reduced cognitive control of the subjects after sleep deprivation found in this study may be caused by the reduction of top-down inhibitory control of the subjects after changes in the activity of the above brain regions, resulting in difficulty in overcoming the dominant response caused by Nogo-food image stimulation (Friedman et al., 2009).

Studies have found that reduced levels of available glucose (Gailliot and Baumeister, 2007; Cullen et al., 2020) and 5-ht (Zimmer et al., 2016) in the brain can impair cognitive control. Since glucose and 5-ht contents in blood are positively correlated with those in cerebrospinal fluid (Audhya et al., 2012), this study reflects changes in the central nervous system after sleep deprivation by detecting glucose and 5-ht contents in peripheral blood. The results showed that there was no significant change in fasting blood glucose content and the content of 5-ht decreased after sleep deprivation, indicating that the decrease of cognitive control ability after sleep deprivation was related to the decrease of central 5-ht content, while glucose may not be the main factor causing the decrease of cognitive control ability. However, the effect of reduced glucose sensitivity on cognitive control after sleep deprivation cannot be ruled out. It has been confirmed that sleep deprivation for one night can reduce insulin sensitivity in the blood (Cedernaes et al., 2016). However, whether reduced insulin sensitivity is the cause of reduced cognitive control in the subjects in this study needs to be further confirmed.

### Physical Exercise Can Alleviate Impaired Cognitive Control After Sleep Deprivation

In this study, we applied a 30-min aerobic exercise to subjects who experienced a night of complete sleep deprivation, and the results showed that compared with the non-exercise group, the subjects had increased key accuracy and shortened response time, significantly decreased ICV, and no significant change in omission error immediately after exercise (0 min) in the face of non-food image stimuli, indicating that exercise improved the subjects' response execution ability. At the same time, commission error was significantly reduced immediately after exercise, indicating that the trend of reduced response inhibition ability of the subjects was reversed after sleep deprivation. In the face of Nogo food image stimulation, the subjects needed top-down inhibitory control to overcome the dominant response (Friedman et al., 2009), and the commission error in the exercise group was lower than that in the rest treatment group in this experiment, indicating that after acute aerobic exercise, the ability of the subjects to flexibly use top-down inhibitory control was improved, reflecting the promoting effect of acute aerobic exercise on inhibitory ability. In addition, the motor response time is also significantly reduced, which shows that the improvement of the correct rate is not at the expense of the response time, but the improvement of the potential inhibitory control ability. The results suggest that the improvement of inhibitory ability by aerobic exercise may inhibit impulsive responses from the time of early perceptual processing. The results are in line with the experimental expectations, and also further confirm the previous studies. The results of the study by Pontifex et al. (2013) showed that after exercise, it was accompanied by an increase in P3 amplitude and an increase in inhibitory control ability. Kashihara and Nakahara (2005) found that moderate-intensity aerobic exercise facilitates the improvement of the speed of selective response in exercisers. Drollette et al. (2014) demonstrated that a moderate intensity exercise could improve response accuracy in children with lower inhibitory control in the flanker task, but the reaction time did not change. Tomporowski (2003) also found that an aerobic exercise of 20–60 min at a time could improve exercisers' performance in cognitive tasks. There are various explanations for the reasons why short-term aerobic exercise improves cognitive control function. The awakening sleep hypothesis suggests that aerobic exercise can increase the awakening level of the body, increase its metabolic level, increase the blood flow level in brain regions related to executive function, and then improve the cognitive function of individuals (Kamijo et al., 2004); the brain-derived neurotrophic factor hypothesis suggests that short-term aerobic exercise increases the neuroendocrine level of the brain, leading to changes in neurotrophic factors in the brain and improving the cognitive function of individuals (Seifert and Secher, 2003). However, the above hypotheses need to be verified by more studies to comprehensively and clearly reveal the mechanism by which short-term aerobic exercise affects cognitive control function. Studies have found that changes in dopamine (Zoladz and Pilc, 2010) and 5-ht (Zimmer et al., 2016) neurotransmitter concentrations in the central nervous system can also affect cognitive function. The results of this experiment also confirmed that the 5-ht neurotransmitter content in the blood of the subjects increased after exercise. Because the prefrontal cortex has a very dense serotonergic innervation, and the prefrontal cortex plays an important role in regulating executive function including response inhibition (Sargin et al., 2019), the increased 5-ht content in the peripheral blood after exercise may increase the 5-ht content in the PFC through the 5-ht transporter (SERT) on the capillaries, which in turn enhances cognitive control (Morici et al., 2022) and reduces impulsivity.

By comparing the comprehensive performance of the subjects in the Go-Nogo task at two time points: 30 min and 1 h after exercise, it was found that exercise had a significant time-course benefit in promoting cognitive control function after sleep deprivation, including cognitive control advantages in both response execution and response inhibition that could be maintained until 1 h after the end of exercise. The reason for this time-course benefit may be that neurotransmitters such as dopamine (Longo et al., 2021) and 5-ht secreted during exercise still play a role in neuromodulation after cessation of exercise, which is speculated to be partially confirmed in the detection of blood 5-ht after exercise. The results of correlation analysis also showed that there was a highly significant positive correlation between hit rate and 5-ht content changes, and a highly significant negative correlation between commission errors and ICV and 5-ht content changes in college students after exercise intervention. The reason for this may be because increased 5-ht content changes the activity of related brain regions such as the prefrontal cortex of the brain, ultimately changing the cognitive resource allocation function of the brain, which is that the response inhibition function and response execution of individuals are enhanced in the face of sensitive signal stimuli. However, this change in cognitive function was not significantly associated with changes in blood glucose content, which is inconsistent with previous studies. In addition, this study found that there was no gender main effect on the promotion of cognitive control ability by aerobic exercise. Because the degree of promotion and time-course benefit of cognitive control by moderate intensity aerobic exercise may be affected by factors such as exercise time, exercise intensity and physical fitness of subjects, therefore, future studies should use different cognitive task paradigms to comprehensively reveal the relationship between motor and cognitive control in terms of single factors, multiple factors and interactions.

## Conclusions

One night of total sleep deprivation impaired cognitive control, including response inhibition and response executive function. Acute, a single bouts of aerobic exercise can alleviate the cognitive control impairment caused by sleep deprivation, and 5-ht may be one of the possible mechanisms by which acute, a single bouts of aerobic exercise alleviates the cognitive control impairment caused by sleep deprivation. This study suggests that aerobic exercise may be an effective emergency way to temporarily alleviate or restore impaired cognitive control ability in special occupations such as truck drivers, soldiers, and nurses who often engage in night shift work when effective sleep is not available.

## Data Availability Statement

The original contributions presented in the study are included in the article/supplementary material, further inquiries can be directed to the corresponding author/s.

## Ethics Statement

The studies involving human participants were reviewed and approved by 2020 Educational Science Planning Project of Shanxi Province (Project Approval No.: J2021729). The patients/participants provided their written informed consent to participate in this study.

## Author Contributions

SL: software, resources, and writing—original draft preparation. RZ: writing—review and editing. All authors have read and agreed to the published version of the manuscript.

## Funding

This work was supported by the 2020 Educational Science Planning Project of Shanxi Province (Project Approval No.: J2021729).

## Conflict of Interest

The authors declare that the research was conducted in the absence of any commercial or financial relationships that could be construed as a potential conflict of interest.

## Publisher's Note

All claims expressed in this article are solely those of the authors and do not necessarily represent those of their affiliated organizations, or those of the publisher, the editors and the reviewers. Any product that may be evaluated in this article, or claim that may be made by its manufacturer, is not guaranteed or endorsed by the publisher.
